# Developing anti-microbial peptide database version 1 to provide comprehensive and exhaustive resource of manually curated AMPs

**DOI:** 10.1038/s41598-023-45016-3

**Published:** 2023-10-19

**Authors:** Rajat Kumar Mondal, Debarup Sen, Ankish Arya, Sintu Kumar Samanta

**Affiliations:** 1 Biochemistry and Bioinformatics Laboratory, Department of Applied Sciences, Indian Institute of Information Technology Allahabad (IIIT-A), Uttar Pradesh, Devghat, Jhalwa, Prayagraj, 211012 India; 2 Persistent Systems Ltd., Pune, Maharashtra India; 3 Department of Applied Sciences, Indian Institute of Information Technology Allahabad, Allahabad, 211012 India

**Keywords:** Protein databases, Molecular medicine

## Abstract

Anti-Microbial Peptide Database version 1 (AMPDB v1) is a meticulously curated resource that aims to address the limitations of existing databases in the field of antimicrobial research. We have utilized the latest technology and put our best efforts into adding all relevant tools to cater to the needs of our users. AMPDB v1 is a derived database, built upon information gathered from the available resources and boasts a significant size of 59,122 entries which are classified into 88 classes. All the information in this resource was curated manually. Sequence alignment and protein feature calculation tools were integrated into the database in the form of web applications, to make them easy to use, quick, and responsive in real-time. We have included multiple types of browsing and searching options to enhance the user experience, from simple text search to a completely customizable advanced search page with intuitive options that let the user combine multiple options together to make a powerful search query. The database is accessible by a web browser at https://bblserver.org.in/ampdb/.

## Introduction

AMPs are small peptides released by a diversity of organisms as a part of their immune defence system. As pathogenic microorganisms, such as bacteria, viruses, fungi, and parasites, are becoming increasingly resistant to multiple drugs, there has been a growing demand for alternative medications in recent decades^1^. In terms of efficacy and potency, a highly recommended alternative can be AMPs, which is a thrust area of research nowadays. AMPs are highly potent against multiple targets like bacteria, virus, fungus, parasites, worms, etc., and inhibit their activity at various levels; due to this property, these peptides are given the name of Anti-Microbial Peptides (AMPs)^1^.

The discovery of the first reported AMP, penicillin, by Alexander Fleming in the 1920s marked a significant milestone^1,2^. Subsequently, in 1939, gramicidin, another AMP, was isolated from *Bacillus spp.*, demonstrating efficacy against pneumococcal infections in mice^1,3,4^. Tyrocidine, the first commercialized AMP, was later discovered but found to be toxic to human blood cells^5^. In 1956, Phagocytin was isolated from rabbit leukocytes^6^. Since 1939, numerous AMPs have been discovered and reported in primary resources such as NCBI databases, EMBL databases, PDB, and literature resources such as PubMed. These discoveries have led to the development of derived databases, including AVPdb^7^, BaAMPs^8^, BACTIBASE^9^, DADP^10^, Defensins KnowledgeBase^11^, HIPdb^12^, InverPep^13^, LAMP^14^, MilkAMP^15^, PenBase^16^, Peptaibols^17^, PhyTAMP^18^, RAPD^19^, SAPdb^20^, YADAMP^21^, ADAM^22^, AMPer^23^, ANTIMIC^24^, APD3^25^, AMSDb^26^, CAMP R4^27^, DBAASP^28^, dbAMP 2.0^29^, DRAMP 3.0^30^, and DAMPD^31^. While some of these resources are actively maintained, others have become obsolete or have been retired due to the inability to keep up with rapidly expanding and evolving data or due to insufficient maintenance. However, it is crucial to acknowledge the wealth of information present in these resources, which remains vital for advanced research in the field of AMPs.

Keeping in mind the above points and to bridge the gaps and shortcomings we have noticed in the existing functioning AMP databases, in this article we present a dedicated generalized resource that we have created for AMPs–titled Anti-Microbial Peptide Database version 1 (AMPDB v1), which is a comprehensive and exhaustive resource of AMPs. AMPDB v1 contains extensive information about 59,122 AMPs from 18,240 unique sources (natural source organism + synthetic construct). The peptides were also classified into 88 different classes based on their activity. The data of this resource has been primarily curated from the NCBI Protein database and EMBL-EBI database. Each entry of AMPDB v1 is annotated into 5 major parts i.e., general descriptions of the peptide, protein sequence and composition, physicochemical properties, activity details, and database cross-references. The database also supports multiple search facilities like quick search, text search, specific text search, physicochemical properties search, protein composition search, peptide activity search, target organism search, advanced search, etc. This resource also comes with 2 toolboxes, the first one for sequence alignment, where a user can do BLASTp search^32^, MSA^33^, PSA (N&W global^34^ and S&W local sequence alignment^35^), and the second one is protein feature calculation toolbox, which a user can use to calculate composition, physicochemical properties, composition transition distribution (CTD) descriptors^36^ & quantitative structure–activity relationship (QSAR) descriptors^37–39^ of protein. Moreover, users can export all the data (including search data and tools outputs) to a local machine for the benefit of their research, in fields such as AMP-based drug design, phylogenetic analysis, statistical analysis, interaction studies, pathways & network studies, etc.

## Materials and methods

### Dataset organisation

#### Data curation and compilation

The primary databases including the NCBI Protein database and EMBL-EBI database were specifically used as source databases and the basic and advanced search facilities of these databases were used to curate the information of AMPDB v1. Certain keywords include ‘antimicrobial’, ‘lantibiotic’, ‘antiviral’, ‘antibiotic’, ‘defensin’, ‘fungicide’, ‘plant defense’, ‘bacteriocins’, ‘amphibian defense peptide’, ‘autophagy’, ‘antibiofilm’, ‘anticandida’, ‘antiparasitic’, ‘antituberculosis’, ‘spermicidal’, and ‘tumor suppressor’ and their combinations were queried to the source databases.

Initially, we got around 15.2 million results in NCBI Protein database and around 3 million results in the EMBL-EBI database for the keyword ‘antimicrobial’ only. In the case of the NCBI Protein database, after the initial search, we used the filter named ‘UniProtKB/Swiss-Prot’ that is present in the NCBI Protein database itself and the search results were reduced to nearly 4700. The advanced search facility was not used here. After this, we build 2 keyword combinations for all keywords (mentioned above) like ‘antimicrobial AND lantibiotic, ‘antimicrobial AND antiviral’, and so on. Those combinations were searched in basic search facility of NCBI Protein database, and used the same filter criteria to retrieve the result(s).

In the case of EMBL-EBI database, after the initial search, we used the advanced search facility where we used the ‘domain’ as ‘UniProtKB’, and in ‘build query’ section we used only ‘antimicrobial’ as ‘keyword’ and searched it. Now the search results were reduced to around 54,000, then the results were retrieved. After that, in advanced search facility, the ‘domain’ remained the same and in ‘build query’ section, the same keyword combinations which were queried in NCBI Protein database, were used in 2 different ways which are following.

- Way1: “keywords:antimicrobial AND keywords:lantibiotic” *[Note: here ‘antimicrobial’ keyword searched in ‘keywords’ AND ‘lantibiotic’ keyword searched in ‘keywords’]*
- Way2: “keywords:antimicrobial AND lantibiotic” *[Note: here ‘antimicrobial’ keyword searched in ‘keywords’ AND ‘lantibiotic’ keyword searched in ‘all fields’ ]*

The above 2 ways were applied for other keywords combinations and were searched followed by result(s) retrieval. For both source database, we used UniProt as filter and domain because it not only narrows down the search result but also help us to curate high-quality and reliable data. Moreover, using a combination of multiple synonymous words like “antimicrobial”, “antiviral”, etc. ensures higher data coverage – an effort to not miss out on entries that may use some other keyword instead of “antimicrobial”.

The data including protein sequence, source organism, gene names, protein names, length of the sequence, peptide existence level, protein families, PubMed ID(s), UniProt ID were manually curated from the retrieved entries to build the master dataset. Before incorporating individual entry in the master dataset, we checked whether the entry already exists or not based on UniProt ID, if exist then the same entry was not incorporated, incorporated otherwise. This way a master dataset was compiled, containing 59,122 various AMPs from 18,240 unique source organisms. All the searches were done & the master dataset was compiled in August–September 2022.

Now we have the UniProt ID in the master dataset. We searched the UniProt ID individually in RCSB-PDB database to get the structure(s) (if found). Likewise, other 25 different databases (including PDBsum, PDBe, PDBj, PDBe-KB, MMDB, AlphaFoldDB, IntAct, STRING, MINT, DIP, BioGRID, BindingDB, ChEMBL, DrugBank, KEGG^40–42^, Ensembl, GeneTree, BRENDA, BioCyc, RNAct, PANTHER, PROSITE, InterPro, CCDS, NCBI RefSeq, etc.) were also searched based on UniProt ID to find whether any extra and exclusive information is available about the AMPs. On finding suitable information, the ID/ACC. No. of the respective entries was noted and added to the master dataset. These noted IDs were used for further database cross-referencing.

#### Calculating protein composition and physicochemical properties

Protein composition and physicochemical properties were calculated by using ProtParam module from Biopython package (version 1.79)^43^, peptides (version 0.3.1)^44^ python package, and our own python package named proteinAnalysis (version 1)^45^.

ProtParam Module of Biopython package (version 1.79)^43^: This is a powerful and widely used collection of Python tools for computational biology and bioinformatics. The ProtParam module specifically provides functionality for analysing protein sequences and calculating various physicochemical properties like amino acid composition and frequency, isoelectric points, etc.

proteinAnalysis(version 1) Python package^45^: It is a very simple Python package that we developed to calculate very basic quantitative information (which includes amino acid counts & frequencies, most & least occurring residues, hydrophobic & hydrophilic amino acid count, basic and acidic amino acid count, modified amino acid count and frequency) of the protein sequences.

Peptides (version 0.3.1) Python package^44^: This is a Python package that only works with protein sequences that computes peptide statistics (like amino acid counts and frequencies) common QSAR descriptors^38,39,43^ (like Cruciani properties, FASGAI vectors, Kidera factors, MS-WHIM scores), sequence profiles (like hydrophobicity, hydrophobic moment), etc.

The table (Table 1) contains a list of functions mentioned from ProtParam Module of Biopython package (version 1.79) ^43^ and proteinAnalysis (version 1) python package ^45^ which were explicitly used for protein composition computation.

**Table 1:** Table of functions used to calculate the protein composition.

Protein Composition Computation			
ProtParam Module of Biopython package (version 1.79)^43^		ProteinAnalysis (version 1)^45^	
Function	Used for	Function	Used for
count_amino_acids	Amino Acid Counts	missingResidues	Missing Amino Acid(s)
get_amino_acids_percent	Frequencies of Amino Acids	mostOccuringResidues	Most Occurring Amino Acid(s)
		lessOccuringResidues	Less Occurring Amino Acid(s)
		hydrophobicAACount	Hydrophobic Amino Acid(s) Count
		hydrophilicAACount	Hydrophilic Amino Acid(s) Count
		acidicAACount	Acidic Amino Acid(s) Count
		basicAACount	Basic Amino Acid(s) Count
		modifiedAACount	Modified Amino Acid(s) Count
		modifiedAAFrequency	Modified Amino Acid(s) Frequencies

The table (Table 2) contains a list of functions shown from ProtParam Module of Biopython package (version 1.79)^43^ and peptides (version 0.3.1) python package^44^ which were explicitly used for protein physicochemical properties computation.

**Table 2:** Table of functions used to calculate the protein physicochemical properties.

Protein Physicochemical Properties Computation			
ProtParam Module of Biopython package (version 1.79)^43^		Peptides (version 0.3.1) python package^44^	
Function	Used for	Function	Used for
instability_index	Instability Index	aliphatic_index	Aliphatic Index
gravy	Hydrophobicity (GRAVY)	hydrophobic_moment	Hydrophobic Moment
isoelectric_point	Isoelectric Point		
molecular_weight	Molecular Weight		
charge_at_pH	Charge (at pH 7)		
secondary_structure_fraction	Secondary Structure Fraction		
aromaticity	Aromaticity		
molar_extinction_coefficient	Molar Extinction Coefficient (cysteine|cystine)		

#### Finding target organism and peptide activity

As mentioned already, we had peptides with their corresponding PubMed Literature ID(s) and UniProt ID in our master dataset. We had gone through the PubMed Literature(s) based on the PubMed Literature ID(s) and UniProt data based on the UniProt ID for the individual peptide to find out various activities (for e.g., antimicrobial activities, enzymatic activities, inhibitory activities, other biological activities, etc.) and target organism of a peptide. On completing the above process, we found 88 unique biological activities and ~ 350 target organisms for 59,122 peptides. Then we merged all this data with our master dataset. At this point, our master dataset became completely ready (in CSV format) for upload to the backend.

#### AMPs classification

The AMPs of the master dataset have been classified into 88 categories based on various activities including enzymatic activity, inhibitory activity, antimicrobial activity, and other relevant biological activity (like anti-cancer, anti-inflammatory, chemotaxis, hemolytic, toxin, etc.). Such classification has been performed based upon and inspired by classification schema as seen in existing databases like APD3^25^, dbAMP 2.0^29^, DRAMP 3.0^30^, etc. However, the classification schema is a little different from other existing AMP databases.

As we mentioned earlier, we found the various activities of AMPs during finding the target organisms. We are discussing the process of activity finding and classification in this section. To find the activity and classify the AMPs, we created 4 columns in our master dataset named (1) ‘antimicrobial activity’, (2) ‘enzymatic activity’, (3) ‘inhibitory activity’, and (4) ‘other biological activity’. By observing the classification from the existing databases like APD3^25^, dbAMP 2.0^29^, DRAMP 3.0^30^, we conceived an idea that the AMPs can also be classified into 4 broad classifications based on their activity. Although there may be other possibilities to classify AMPs, but we choose 4 broad class-based classification because none of the existing resources offers this kind of classification. Hence, we created those 4 columns. Other than our master dataset, we maintained another Excel document named ‘classification’ where we created the same 4 columns with 2 sub-columns namely (1) ‘activity’, and (2) ‘total entries’. After that, we started reviewing the activity in the PubMed literature and UniProt data for each entry. On finding each activity for the respective entry, we incorporated the activity information in the respective broad class of the master dataset. Along with this, in the respective broad class in ‘classification’ document, we incorporated that activity information in the ‘activity’ sub-column and increased the count by + 1 in ‘total entries’ sub-column (if an activity is found for the first time), and if the same activity is found for another entry, then we updated the count by + 1 in same ‘total entries’ sub-column only. After completion of this process, we got all activity details under the respective broad class for each entry in the master dataset, and along with this, we got the ‘classification’ file which contains unique activities in the ‘activity’ column with their count in ‘total entries’ column under their respective broad class. The data of the ‘classification’ file is provided in the supplementary Table S1. After the classification, we have a total of 89 datasets (i.e., 88 datasets based on activity + the AMPDB v1 master dataset), which were finally stored at the backend.

### Backend and frontend development

Backend and frontend development was done by using cross-platform and open-source language(s).

#### Database architecture of the backend

AMPDB backend is built on HTTPS server with PhpMyAdmin (version 5.1.1) and inside that MySQL server (Relational Database Management System (RDBMS)) (version 5.7.36). All 89 datasets were stored inside the MySQL server.

#### Frontend development

HTML5, CSS3, Bootstrap5, JavaScript, AJAX, jQuery, DataTables, Chart.js, etc. were used for frontend development. Frontend was connected to the backend using PHP. SQL is used inside the PHP scripts for queries in the database via the frontend. The database also supports server-side processing (lazy loading) to return large chunks of data to the user in a very minimalistic time.

### Tools development

Currently, 8 tools are integrated into AMPDB v1. The tools are written in pure Python (version 3.10) language^46^ by using some open source non-pythonic modules/programs and Python packages. The First 4 tools (including BLASTp^35^, multiple sequence alignment (MSA)^36^, pairwise sequence alignment (PSA) (N&W global alignment^34^, and S&W local alignment^35^)) are for sequence alignment. For the developed BLASTp search facility, the NCBI BLAST+ module^47^ was used and for the MSA facility, the MUSCLE module^48^ was used. For N&W global alignment, S&W local alignment “local_pairwise_align_protein” and “global_pairwise_align_protein” functions from the alignment module of scikit-bio (version 0.5.8) python package^49^ are used. First, 4 alignment tools are wrapped up into a toolbox named AMPDB sequence alignment toolbox.

The other 4 tools (including protein sequence composition computation, protein physicochemical properties computation, CTD descriptors^36^ computation, and QSAR descriptors^37–39^ computation) are integrated for protein features calculation. Protein Analysis (version 1)^45^ python package and ProtParam module from Biopython package^43^ are used to develop protein sequence composition & physicochemical properties computation facility. For CTD descriptors and QSAR descriptor computation facility, propy3^50^ and peptides (version 0.3.1)^44^ Python packages are used respectively. These 4 tools are also wrapped up in a toolbox named AMPDB protein feature calculation toolbox.

Streamlit python package^51,52^ was used specifically used for designing the interactive UI of the tools.

### User interface of the database

**Figure 1:**
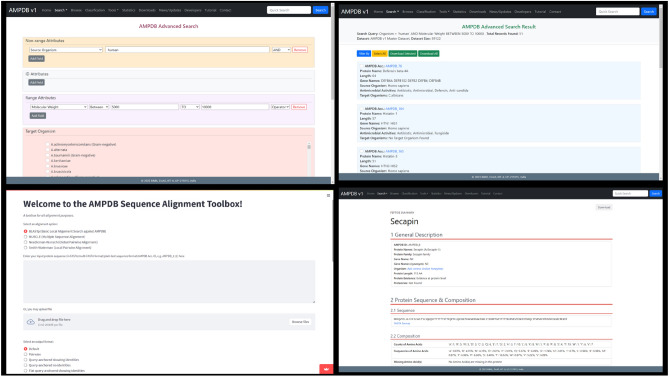
Some screenshots of the user interface of AMPDB v1.

A minimalistic UI (shown in Fig. 1) has been provided to interact (includes searching, export, visualization, protein sequence alignment, protein sequence feature calculation, etc.) with the data of AMPDB v1. It has been kept as user-friendly as possible.

#### Home

In this section, there is a very brief description of the database and along with various options. A quick search option is provided for querying any term in the database.

#### Search

Currently, 8 types of major search features are provided by AMPDB v1, which are following.

##### Quick search

By using this facility user can search any term related to AMPs without going to other search options. This facility is provided on every page of the UI.

##### Text search

This generalized search facility of AMPDB v1 enables the user to query any term related to AMPs in the database.

##### Specific text search

**Figure 2:**
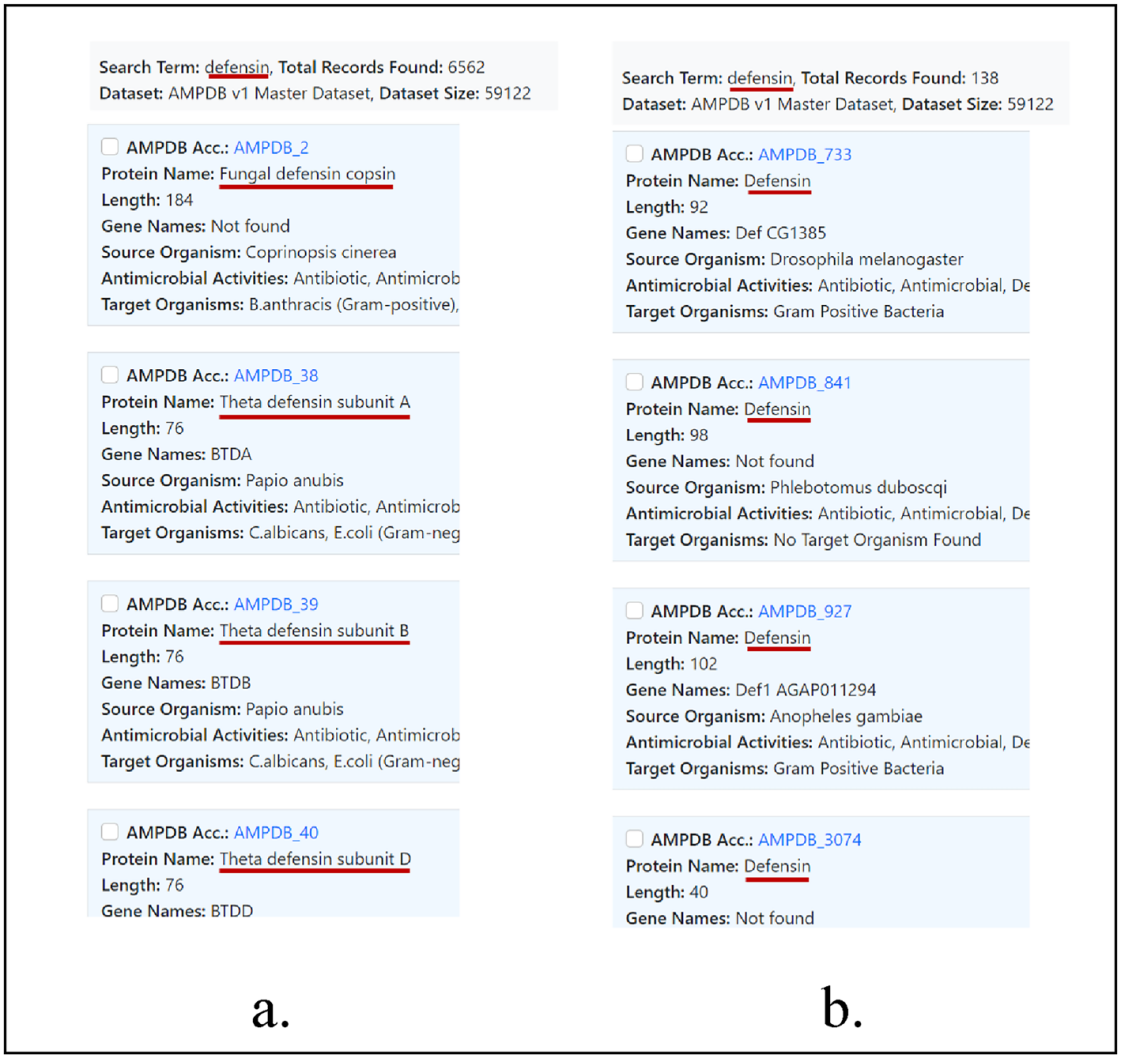
Comparison between text search and specific text search. a. Text search b. Specific text search. In both cases search the search term and results are highlighted with a dark red underline.

This search facility is similar to the ‘text search’ facility. Users can search using any term related to AMPs. The main difference is that it shows only those results which have exactly matched the search term. A comparative image is shown in Fig. 2.

##### Search by target organism

Target organism search facility has been specifically designed to query in the database with a single or multiple target organism’s name(s).

##### Search by protein physicochemical properties

This search enables the user to build the search query by just selecting the options(s) (like Molecular Weight, Charge, Isoelectric Point, etc.) as needed. The user can build search queries by various operators (like AND, OR, NOT, BETWEEN, Not Eq, >, <, >=, <=) along with range or exact value.

##### Search by protein composition

In this search facility user can build the query by selecting the protein composition option(s) (like Protein Length, Most Occurring Amino Acid(s), Less Occurring Amino Acid(s) etc.) with operators and range or exact value.

##### Search by activity

In this section various activities (shown in Supplementary Table S1) are listed, which are found in AMPs. Users can select single or multiple checkbox(s) as per their interest for search.

##### Advanced search

The advanced search section brings together all the aforementioned facilities into one-fold, in an attempt to provide a powerful platform that enables the user to club multiple parameters and filters to narrow down their search to absolutely specific ranges.

By default, all the searches will be done against AMPDB v1 master dataset. User also can restrict their search by clicking on the ‘Choose a Dataset’ option in UI to restrict their search against a particular dataset out of 88 datasets.

All the search results appear in card format. Various filters can be used to narrow down the results. The results can be downloaded in TSV, FASTA, TEXT, XML, JSON, LIST formats, and among these formats TSV, JSON, and XML are completely customizable. Each entry can be viewed individually by clicking on the AMPDB accession and can be downloaded further in TEXT, FASTA, and PDF format.

#### Browse

In this section users can browse all the entries of the database.

#### Classification

It has already been discussed that 88 different classifications (shown in Supplementary Table S1) had been identified. All the classification data has been enlisted in this section. Users can view all entries of a particular classification by just clicking on the hyperlink.

#### Tools

It is already mentioned that AMPDB v1 has two toolboxes. In the AMPDB sequence alignment toolbox user can do the following,

- **BLASTp: **BLASTp can be done against either of the datasets of AMPDB v1. We have provided the option of 14 output formats, including default, Pairwise, Query-anchored, and Flat query-anchored formats with or without identities, Tabular, XML, ASN, and JSON formats as well. For specific results based on specific parameters, we have provided options to determine parameters like dataset, e-value, query coverage, word size, minimum word score, gap open and gap extend penalties, choice of matrices, and much more.
- **MSA by using MUSCLE: **Multiple sequence alignment of multiple AMPs or other peptides can be done by this tool. This tool also allows the choice of parameters regarding whether to find diagonals, the maximum number of iterations, the maximum number of hours to iterate, and the choice of formats including HTML, GCG MSF, and ClustalW (with or without header line), and whether to output sequences in input order or group them by similarity.
- **Pairwise sequence alignments: **Global and local sequence alignment of multiple AMPs or other peptides can be done by this tool. The Needleman-Wunsch global alignment method and Smith-Waterman local alignment methods provide options to enter gap open and gap extension penalties. The result also can be exported further in text or other formats.

The protein feature calculation toolbox provides the options for calculation of protein sequence composition, physicochemical properties, CTD descriptors as well as QSAR descriptors. All results can be exported further in text or CSV format.

#### Statistics

**Figure 3:**
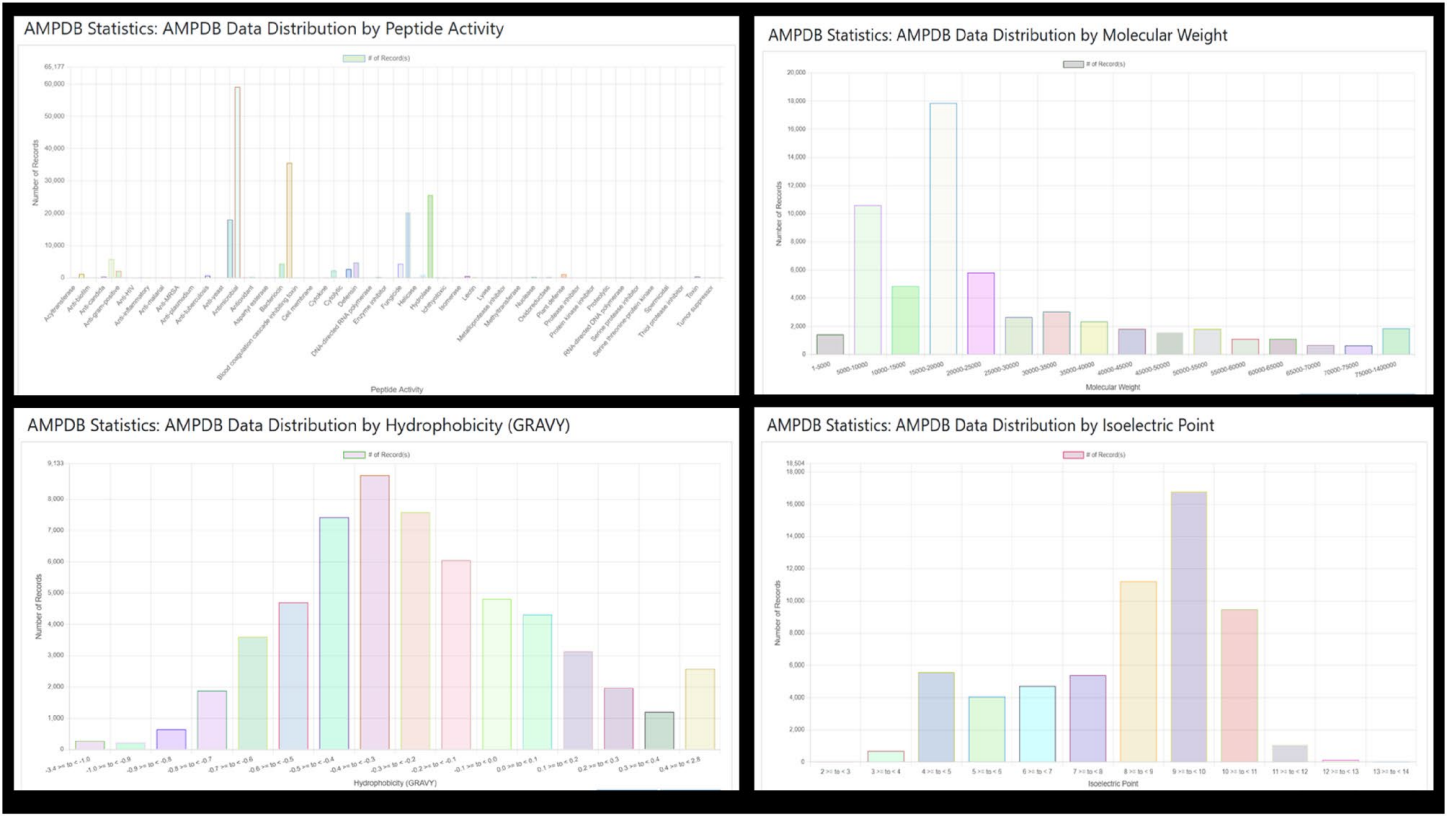
AMPDB v1 data statistics of few categories.

The data statistics of AMPDB have been shown in 14 different categories which include data statistics based on Activity, Residue Length, Molecular Weight, Aliphatic Index, Instability Index, Hydrophobicity (GRAVY), Hydrophobic Moment, Isoelectric Point, Charge (at pH 7), Aromaticity, Hydrophobic Amino Acid Count, Hydrophilic Amino Acid Count, Basic Amino Acid Count, Acidic Amino Acid Count. The initial scale is set with 10 ticks on the Y-axis. Because the difference between the lowest number of records and the highest number of records is very high, therefore, the graph is designed in this way for better visualization. Users can click on the "Rescale X-Axis" or "Rescale Y-Axis" button to rescale the plot. Some of the data statistic graphs are shown in Fig. 3.

#### Downloads

All the datasets of AMPDB v1 can be downloaded from here in TSV, TEXT, FASTA, LIST, and JSON format.

#### News/updates

New and latest features/updates about this database or AMPs will be posted in this section. The latest 2 news (sorted by date) will be shown on the homepage from here.

#### Developers

Details about the developers of AMPDB have been shown in this page.

#### Contact section

Contact details of the principle investigator and developers have been provided here. All comments and suggestions are welcome. Users can contact us by given mail addresses or fill out the web-form.

## Results

AMPDB v1 is a comprehensive and exhaustive resource on widely found AMPs. Currently, the AMPDB v1 holds 59,122 peptides from 18,240 source organisms. The peptide can be used to target ~ 350 target organisms. The peptides were classified into 88 different classes based on their activity (shown in Supplementary Table S1), this will benefit researchers to study a particular class of AMPs. The 88 different classes were treated as 88 different datasets so that a user can restrict their search against one of the datasets as per interest during query in the database or BLASTp search, AMPDB v1 master dataset is default otherwise.

Currently, 8 types of search features (excluding BLASTp) are provided in this database, so that researchers can query the database as per interest. Server-side processing is implemented for faster response to each query. All the search results will appear as cards and can be filtered based on source organism, anti-microbial activity, target organism, sequence length, protein existence level, and can be sorted, in ascending or descending order, by protein name, gene name, sequence length, etc. and can be previewed as well as downloaded on the local machine in various file formats for further study. For each individual entry, the database displays information in 5 different parts which are the following,

1. General description of the peptide (includes AMPDB ID, peptide name and family, source organism, gene name, peptide existence level, peptide length).
2. Protein sequence and composition (includes the sequence of the protein, counts of amino acids, frequencies of amino acids, missing amino acid(s), most occurring amino acid(s), less occurring amino acid(s), hydrophobic amino acid(s) count, hydrophilic amino acid(s) count, basic amino acid(s) count, acidic amino acid(s) count, modified amino acid(s) count, modified amino acid(s) frequencies).
3. Physicochemical properties (includes molecular mass, aliphatic index, instability index (half-life), hydrophobicity (GRAVY), hydrophobic moment, isoelectric point, charge (at pH 7), secondary structure fraction, aromaticity, molar extinction coefficient (cysteine|cystine)).
4. Activity details (includes target organism, antimicrobial activity, enzymatic activity, inhibitory effect, and other biological activity).
5. Database cross references (contains other databases ID(s) (like PubMed, UniProt, GenBank, RCSB-PDB & 26 more as hyperlinks). In the ‘3D Structure Databases’ subsection of this database cross-references section 3Dmol.js^53,54 ^was integrated to visualize the 3D structure of the peptide (if found). Users can view the 3D structure by just clicking on the ‘View’ option.

Researchers also can export the data of individual entries further for studies. There can be multiple use cases possible for AMPDB v1, 2 are shown below.

**Use case 1:** Designing novel AMPs

*Background*: A researcher wants to design a novel AMP with improved antimicrobial activity, for which the researcher decides to explore existing AMPs in AMPDB v1 to identify sequence motifs and physicochemical properties associated with target specific AMPs.

The provided steps can be followed to fulfill use case 1:

1. *Querying specific AMPs*: The researcher starts by using the search functionalities in AMPDB v1 to search and filter for AMPs with specific antimicrobial activity, based on classification, target organism, or any other relevant criteria.
2. *Analysis of sequence motifs*: After obtaining a list of appropriate AMPs, the researcher uses the protein sequence and compositional data provided by AMPDB v1 to identify common sequence motifs present among these peptides, that may be associated with antimicrobial activity.
3. *Physicochemical properties analysis*: The researcher also examines the physicochemical properties of these AMPs, such as hydrophobicity, isoelectric point, and molecular mass, to find out the range of values for each of these features.
4. *Rational design*: The researcher may use the insights obtained from above mentioned analyses to design a novel AMP by incorporating the identified sequence motifs and optimizing physicochemical properties to enhance antimicrobial activity.
5. *Experimental validation*: The novel AMP can be experimentally tested in vitro or in vivo to evaluate its antimicrobial efficacy. If successful, this novel AMP could potentially serve as a new antimicrobial agent.

**Use case 2:** Understanding AMP-target interactions

*Background*: A researcher aims to deepen the understanding of how AMPs interact with microbial targets at the molecular level. The researcher wants to study the binding affinities and structural characteristics of AMPs in complexes with target organisms.

The provided steps can be followed to fulfill use case 2:

1. *Select target organism*: The researcher begins by selecting a specific target organism of interest, such as *Candida sp.*
2. *Retrieve relevant AMPs*: Searching through AMPDB v1, the researcher retrieves a list of relevant AMPs by using target organism filters.
3. *Retrieve structural information*: The researcher may use cross-references to protein structure databases (such as RCSB-PDB) present in AMPDB v1 to access the structural data for the selected AMPs (if available, AlphaFoldDB can be use otherwise).
4. *Molecular docking*: Using molecular docking software, the researcher may then perform molecular docking and simulation studies to observe the binding modes and affinities between the selected AMPs and the target proteins in *Candida sp*.
5. *Analysing binding interfaces*: The researcher then analyses the binding interfaces and molecular interactions to understand the mode of action of the AMPs.
6. *Structural insights*: Insights gained from molecular docking and structural analysis can help in the design of more effective AMPs and can lead to the development of new strategies to combat drug-resistant microbes.

### Comparison with other databases

To date, on AMPs, two types of databases have been developed i.e., specialized and generalized. AVPdb^7^, BaAMPs^8^, BACTIBASE^9^, DADP^10^, Defensins KnowledgeBase^11^, HIPdb^12^, InverPep^13^, LAMP^14^, MilkAMP^15^, PenBase^16^, Peptaibols^17^, PhyTAMP^18^, RAPD^19^, SAPdb^20^, YADAMP^21^, and AMSDb^26^, these specialized databases of AMPs were dedicated to a very specific or special type of AMPs.

AMSdb^26^, PhytAMP^18^, InverPep^13^, BACTIBASE^9^, PenBase^16^, and MilkAMP^15^ are databases of AMPs from eukaryotes, plants, invertebrates, bacteria, shrimps, and milk. AVPdb^7^ and HIPdb^12^ are dedicated to those AMPs which can be used to target viruses and HIV specifically whereas YADAMP^21^ deals with those AMPs which are used to target only bacteria. The RAPD^19^ and SAPdb^17^ databases hold data about recombinant and synthetic AMPs. BaAMPs^8^ and DADP^10^ have information about biofilm active peptides and anuran defense peptides whereas LAMP^14^ contains information about linking AMPs. Defensins KnowledgeBase^11^ and Peptaibols^17^ contain data about various defensins and peptaibols respectively. Some of these databases are unaccusable and some are maintained to date.

There are also very well-known generalized databases on AMPs like ADAM^22^, AMPer^23^, ANTIMIC^24^, APD3^25^, CAMP R4^27^, DBAASP^28^, dbAMP 2.0^29^, DRAMP 3.0^30^, DAMPD^31^. These databases hold more general data on AMPs. Among the generalized databases AMPer^23^, ANTIMIC^24^, and DAMPD^31^ are retired and others are on-track. We have gone through these databases and have found, that in terms of size of the database, AMPDB v1 contains a much higher number of records (~ 60,000) and provides much more information per record than is currently available in any of the databases. Additionally, we have added newer search features in AMPDB v1, providing the ability to build highly customized queries for data extraction and manipulation while being fully interactive and user friendly; showing all information as cards. AMPDB v1 offers minutely documented interactive help pages to aid users in utilizing the full potential of the database. We have made AMPDB v1 unique in including protein physicochemical and compositional data as well as providing options to query the database by these properties and protein activities. Moreover, AMPDB v1 provides extensive cross-referencing with the major protein sequence, structure, and literature databases; also providing the facility to search by cross-references.

We are also the first in providing certain features, like QSAR and CTD descriptor calculation, which we have felt to be of crucial significance to various types of research work. Altogether, while developing AMPDB v1, we have tried our best to improve upon features that the on-track databases provided and include those features which we as researchers expected from a database but could not find in any. We admit that our work is far from over. AMPDB v1 still lacks certain important information that we have found in current online databases, for example, MIC information of target organisms and post-translational modification information of the AMPs, which we are currently in the process of curating, and expect to add by the next version. Along with that, we are also working on the development of a state-of-the-art AMP prediction tool, which will also be ready by the next version. A comparative study between on-track databases and AMPDB v1 is provided in Supplementary Tables S2 and S3.

AMPDB v1 holds as much as information possible and is developed in such a way as to understand the data of this resource very easily. Along with this, we have tried to provide all facilities which should be available in a peptide-related database. The database aids in drug design, phylogenetic analysis, statistical modelling, studying AMP-target interactions, exploring AMPs for wound healing and tissue regeneration, and more. AMPDB v1 is a valuable resource for AMP research with its comprehensive data, user-friendly interface, and diverse applications.

## Discussion

AMPDB v1 opens a wide horizon of research to the scientific community. The comparison of user-defined query sequences against existing AMPs can assist in mutation studies between the query sequence and existing AMPs, researchers also can design the mutation in the query sequence that could improve the query sequence's antibacterial activity^27^. By this, it is possible to do the rational design of AMPs, which are great substitutes for current antimicrobial agents^27^. For this BLASTp is incorporated, by using that researchers can do a BLASTp search against any of the datasets. Multiple sequence alignment (MSA) studies between AMPs play a very important role in the identification of homologous sequences, detection of conserved regions, prediction of AMP structure and function, evolutionary analysis, phylogenetic studies, and discovery of sequence motifs, etc.^33,55,56^. For that MUSCLE^33^ is provided, so that researchers can do studies on the MSA of AMPs. Needleman-Wunsch pairwise global alignment is incorporated for the study of alignment between two AMPs, which allows a researcher to compare a pair of distantly related sequences of AMPs, aids in structural and functional analysis, and facilitates evolutionary studies ^34^. Smith-Waterman pairwise local alignment also provided for the study of the alignment between a pair of AMPs, which allows for the identification of local similarities, offers increased sensitivity in detecting subtle similarities, aids in the identification of functional domains and motifs, and provides structural and functional insights into specific regions of sequences^57,58^. All the sequence alignment features are available to the researchers in the form of a toolbox named the AMPDB sequence alignment toolbox.

The protein sequence composition and physicochemical properties data computation facility also enables a researcher to compute the information about protein sequence composition and physicochemical properties of any AMPs or other peptides in real-time, by using that information a researcher will be able to get insights into protein structure, function, evolution, and interactions. Researchers can also use that information for classifying AMPs or other peptides into families, understanding functional conservation and diversification, and understanding the drug-likeliness of an AMP. AMPDB v1 also facilitates protein sequence CTD descriptors and QSAR descriptors computation feature as well. A researcher can compute those descriptors of AMPs and use that information for capturing important sequence features, protein function prediction, subcellular localization, structural disorder prediction, protein–protein interaction prediction, and secondary structure prediction. All these computation features are also wrapped up in a toolbox named the AMPDB protein feature calculation toolbox.

There is no limit on protein sequence length but the upper limit is 200 MB in the case of both toolboxes when a file will upload. The toolboxes are hosted by the Streamlit community cloud so that a user gets faster and more real-time responses.

AMPDB v1 was built with effort and passion, driven by the foresight that in the rising tide of microbial resistance against synthetic drugs, AMPs have the potential to become the medicines of tomorrow. To put forth our passion to serve the scientific community in the most helpful way, we have tried our best to include as much information as possible and design the database with as much intuitiveness as possible. When accessing a database, we anticipate meticulous curation, extensive cross-referencing, robust search and filtering capabilities, seamless integration with tools, and an implicit assurance that the database will be available whenever we require it. AMPDB v1 not only fulfils these expectations but goes above and beyond, and we extend a warm invitation to our fellow scientists to explore its offerings.

We humbly present this resource to all scientists and researchers, with the hope that it may aid in furthering their research. We are continuously reviewing the literature, patents, etc. in order to include information about more AMPs in the next version of AMPDB. We are simultaneously working to build more cutting-edge tools to promote AMP-related research.

## Availability of the resource

AMPDB v1 is designed, developed, and maintained by the Biochemistry & Bioinformatics Laboratory, Department of Applied Sciences, Indian Institute of Information Technology Allahabad (IIIT-A), Devghat, Jhalwa, Prayagraj-211012, Uttar Pradesh, India. The resources can be accessed via the web at the URL: https://bblserver.org.in/ampdb/ would like to invite all academicians, researchers, and individuals working in pharmaceutical industries to visit and use this resource.

## Availability of the datasets

All the datasets of this resource are publicly available in JSON, TSV, TEXT, FASTA, and LIST format at the URL: https://bblserver.org.in/ampdb/ampdb-downloads.

### Supplementary Information

Supplementary Tables.

## Data Availability

All the customized codes which are used to develop the UI and tools of AMPDB v1 are publicly available at the URL: https://bblserver.org.in/ampdb/ampdb-downloads (bottom portion of the page). The codes also can be found directly in GitHub at the following link: Website Codes Availability (Except Tools): https://github.com/rajat-kumar-mondal/ampdb_v1. Codes Availability (Tools Only): https://github.com/Debarup-Sen/repoOneAMPDB generate protein composition (includes the missing amino acid(s), most occurring amino acid(s), less occurring amino acid(s), hydrophobic amino acid(s) count, hydrophilic amino acid(s) count, basic amino acid(s) count, acidic amino acid(s) count, modified amino acid(s) count, modified amino acid(s) frequencies) in the datasets we used a custom python package named *proteinAnalysis (version 1)*, that we developed exclusively. The python package is publicly available at GitHub URL: https://github.com/rajat-kumar-mondal/proteinAnalysis. Except the above-mentioned custom codes, no other custom codes were used.
