# Supplementary material for: Developing anti-microbial peptide database version 1 to provide comprehensive and exhaustive resource of manually curated AMPs
Source: Sci Rep. 2023 Oct 19;13:17843. doi: 10.1038/s41598-023-45016-3 (PMC10587344; doi:10.1038/s41598-023-45016-3)
Supplement: Supplementary file 1 — Supplementary Tables. [file 41598_2023_45016_MOESM1_ESM.docx]

**Developing Anti-Microbial Peptide Database Version 1 to Provide Comprehensive and Exhaustive Resource of Manually Curated AMPs**

Rajat Kumar Mondal^1a^, Debarup Sen^1b^, Ankish Arya^2^, Sintu Kumar Samanta^1^*

^1a,2,1*^Biochemistry & Bioinformatics Laboratory, Department of Applied Sciences, Indian Institute of Information Technology Allaha​bad (IIIT-A), Devghat, Jhalwa, Prayagraj-211015, Uttar Pradesh, India.

^1b^Domain Engineer, Persistent Systems Ltd., Pune, Maharashtra, India

**Supplementary Data**

**Table S1**. Classification of AMPs in AMPDB v1.

| **Antimicrobial Activities** | | **Enzymatic Activities** | | **Inhibitory Activities** | | **Other Biological Activities** | |
| --- | --- | --- | --- | --- | --- | --- | --- |
| **Activity** | **Total Entries** | **Activity** | **Total Entries** | **Activity** | **Total Entries** | **Activity** | **Total Entries** |
| Amphibian defence peptide | 1187 | Acyltransferase | 1 | Blood coagulation cascade inhibiting toxin | 9 | Anti-cancer | 51 |
| Anti-HSV | 11 | Aspartyl esterase | 1 | DNA replication inhibitor | 2 | Anti-inflammatory | 17 |
| Anti-MRSA | 80 | Carboxypeptidase | 1 | Metalloenzyme inhibitor | 5 | Anti-uterotonic | 1 |
| Anti-biofilm | 51 | DNA-directed RNA polymerase | 3 | Metalloprotease inhibitor | 5 | Antinociceptive | 1 |
| Anti-candida | 420 | Endonuclease | 267 | Platelet aggregation inhibiting toxin | 16 | Autophagy | 9 |
| Anti-gram-Positive | 2238 | Exonuclease | 3 | Protease inhibitor | 100 | Chemotaxis | 32 |
| Anti-gram-negative | 5800 | Glycosidase | 20322 | Protein kinase inhibitor | 1 | Cytokine | 11 |
| Anti-hepatitis | 2 | Helicase | 2 | Protein synthesis inhibitor | 7 | Cytolysis | 2329 |
| Anti-listeria | 31 | Hydrolase | 25578 | Serine protease inhibitor | 91 | Cytolytic | 52 |
| Anti-malarial | 2 | Isomerase | 2 | Thiol protease inhibitor | 6 | Cytotoxin | 2727 |
| Anti-mollicute | 3 | Ligase | 2 |  |  | Enzyme inhibitor | 101 |
| Anti-parasitic | 100 | Lyase | 162 |  |  | Haemolytic | 867 |
| Anti-plasmodium | 7 | Metalloprotease | 5 |  |  | Hypotensive | 2 |
| Anti-protozoal | 40 | Methyltransferase | 3 |  |  | Ichthyotoxic | 7 |
| Anti-tuberculosis | 744 | Nuclease | 272 |  |  | Insecticidal | 62 |
| Anti-yeast | 79 | Nucleotidyltransferase | 5 |  |  | Proteolytic | 13 |
| Antibiotic | 18067 | Oxidoreductase | 262 |  |  | Signal peptide | 94 |
| Antimicrobial | 59122 | Peroxidase | 70 |  |  | Spermicidal | 17 |
| Antioxidant | 356 | Protease | 114 |  |  | Synergistic peptide | 22 |
| Antiviral protein | 33 | RNA-directed DNA polymerase | 3 |  |  | Tumour suppressor | 1 |
| Bacteriocin | 4411 | Rotamase | 1 |  |  | Wound healing | 6 |
| Bacteriolytic enzyme | 35630 | Serine protease | 15 |  |  |  |  |
| Defensin | 4726 | Serine threonine-protein kinase | 11 |  |  |  |  |
| Fungicide | 4391 | Thiol protease | 79 |  |  |  |  |
| Lantibiotic | 530 | Transferase | 50 |  |  |  |  |
| Plant defence | 1158 |  |  |  |  |  |  |

Table S1 contains 4 broad classes of AMP’s activities i.e., antimicrobial activities, enzymatic activities, inhibitory activities, and other biological activities. Under every broad class, the relative classes and their total number of entries are enlisted.

**Table S2.** A comparative study on the between on-track databases and AMPDB v1.

| **Points** | **Sub-points** | **APD3** | **ADAM** | **DBAASP** | **DRAMP** | **CAMP** | **dbAMP** | **AMPDB v1** |
| --- | --- | --- | --- | --- | --- | --- | --- | --- |
| Version | Current Version | 3 | 3 | 3 | 3 | 4 | 2 | 1 |
| Size | Total number of Records | 3569 | 7811 | 20638 | 22499 | 24243 | 26447 | 59122 |
| Classification | Total number of classifications | 25 | 0 | 0 | 6 | 5 | 53 | 88 |

Table S2 contains the comparison of database version, total number of records, and total number of classifications. Red colour indicates the lowest number and green colour indicates the highest number. Intermediate numbers are indicated by intermediate colours between red and green.

**Table S3.** A comparative study on data annotation and tools between on-track databases and AMPDB v1.

| **Points** | **Sub-points** | **APD3** | **ADAM** | **DBAASP** | **DRAMP** | **CAMP** | **dbAMP** | **AMPDB v1** |
| --- | --- | --- | --- | --- | --- | --- | --- | --- |
| Data Annotation | Protein Names | 1 | 1 | 1 | 1 | 1 | 1 | 1 |
|  | Protein Family | 1 | 1 | 0 | 1 | 1 | 0 | 1 |
|  | Gene Name | 0 | 0 | 0 | 1 | 0 | 0 | 1 |
|  | Synonym | 0 | 0 | 0 | 0 | 0 | 0 | 1 |
|  | Source Organism | 1 | 1 | 0 | 1 | 1 | 1 | 1 |
|  | Protein sequence/Length | 1 | 1 | 1 | 1 | 1 | 1 | 1 |
|  | Protein Existence | 0 | 0 | 0 | 1 | 1 | 1 | 1 |
|  | Proteomes | 0 | 0 | 0 | 0 | 0 | 0 | 1 |
|  | Protein Composition Data | 1 | 1 | 0 | 1 | 0 | 0 | 1 |
|  | Physicochemical data | 1 | 1 | 0 | 1 | 0 | 1 | 1 |
|  | Target organisms | 1 | 0 | 1 | 1 | 1 | 1 | 1 |
|  | MIC value for target organisms | 1 | 0 | 1 | 1 | 1 | 1 | 0 |
|  | Structure | 1 | 1 | 1 | 1 | 1 | 1 | 1 |
|  | Antimicrobial & other biological activity | 1 | 1 | 1 | 1 | 1 | 1 | 1 |
|  | Haemolytic activity | 0 | 0 | 0 | 0 | 1 | 0 | 1 |
|  | Post-translational modifications | 1 | 0 | 0 | 0 | 0 | 0 | 0 |
|  | Binding target | 0 | 0 | 0 | 1 | 0 | 1 | 1 |
|  | Reference | 1 | 1 | 1 | 1 | 1 | 1 | 1 |
|  | UniProt ID | 1 | 1 | 1 | 1 | 1 | 1 | 1 |
|  | PDB ID | 1 | 1 | 1 | 1 | 1 | 1 | 1 |
|  | MMDB ID | 0 | 0 | 0 | 0 | 0 | 0 | 1 |
|  | AlphaFoldDB ID | 0 | 0 | 0 | 0 | 0 | 0 | 1 |
|  | GenBank ID | 0 | 0 | 0 | 0 | 0 | 0 | 1 |
|  | EMBL0Bank ID | 0 | 0 | 0 | 0 | 0 | 0 | 1 |
|  | CCDS ID | 0 | 0 | 0 | 0 | 0 | 0 | 1 |
|  | NCBI RefSeq ID | 0 | 0 | 0 | 0 | 0 | 0 | 1 |
|  | STRING ID | 0 | 0 | 0 | 0 | 0 | 0 | 1 |
|  | IntAct ID | 0 | 0 | 0 | 0 | 0 | 0 | 1 |
|  | MINT ID | 0 | 0 | 0 | 0 | 0 | 0 | 1 |
|  | DIP ID | 0 | 0 | 0 | 0 | 0 | 0 | 1 |
|  | BioGRID ID | 0 | 0 | 0 | 0 | 0 | 0 | 1 |
|  | DrugBank ID | 0 | 0 | 0 | 0 | 0 | 0 | 1 |
|  | ChEMBL ID | 0 | 0 | 0 | 0 | 0 | 0 | 1 |
|  | InterPro ID | 0 | 0 | 0 | 0 | 0 | 0 | 1 |
|  | PANTHER ID | 0 | 0 | 0 | 0 | 0 | 0 | 1 |
|  | PROSITE ID | 0 | 0 | 0 | 0 | 0 | 0 | 1 |
|  | Ensembl ID | 0 | 0 | 0 | 0 | 0 | 0 | 1 |
|  | KEGG ID | 0 | 0 | 0 | 0 | 0 | 0 | 1 |
|  | GeneTree ID | 0 | 0 | 0 | 0 | 0 | 0 | 1 |
|  | BRENDA ID | 0 | 0 | 0 | 0 | 0 | 0 | 1 |
|  | BioCyc ID | 0 | 0 | 0 | 0 | 0 | 0 | 1 |
|  | RNAct ID | 0 | 0 | 0 | 0 | 0 | 0 | 1 |
| Tools | BLAST | 1 | 0 | 0 | 1 | 1 | 1 | 1 |
|  | Multiple Sequence Alignment | 1 | 0 | 0 | 1 | 1 | 0 | 1 |
|  | Pairwise Global Sequence Alignment | 0 | 0 | 0 | 1 | 0 | 0 | 1 |
|  | Pairwise Local Sequence Alignment | 0 | 0 | 0 | 1 | 0 | 0 | 1 |
|  | Protein Composition Computation | 1 | 0 | 0 | 0 | 1 | 0 | 1 |
|  | Protein Physicochemical Properties Computation | 1 | 0 | 1 | 0 | 1 | 0 | 1 |
|  | Protein CTD Descriptors Computation | 0 | 0 | 0 | 0 | 0 | 0 | 1 |
|  | Protein QSAR Descriptors Computation | 0 | 0 | 0 | 0 | 0 | 0 | 1 |
|  | AMP Prediction | 1 | 1 | 1 | 1 | 1 | 1 | 0 |

In TableS3, 1 (represented with green colour) implies the presence of the annotation or tool and 0 (represented with red colour) implies the absence of the annotation or tool.
